# The factors that favor adaptive habitat construction versus non‐adaptive environmental conditioning

**DOI:** 10.1002/ece3.8763

**Published:** 2022-03-24

**Authors:** Samuel M. Scheiner, Michael Barfield, Robert D. Holt

**Affiliations:** ^1^ Division of Environmental Biology National Science Foundation Alexandria Virginia USA; ^2^ Department of Biology University of Florida Gainesville Florida USA

**Keywords:** constitutive theory, environmental structure, habitat construction, kin selection, model, niche construction

## Abstract

Adaptive habitat construction is a process by which individuals alter their environment so as to increase their (inclusive) fitness. Such alterations are a subset of the myriad ways that individuals condition their environment. We present an individual‐based model of habitat construction to explore what factors might favor selection when the benefits of environmental alterations are shared by individuals of the same species. Our results confirm the predictions of inclusive fitness and group selection theory and expectations based on previous models that construction will be more favored when its benefits are more likely to be directed to self or near kin. We found that temporal variation had no effect on the evolution of construction. For spatial heterogeneity, construction was disfavored when the spatial pattern of movement did not match the spatial pattern of environmental heterogeneity, especially when there was spatial heterogeneity in the optimal amount of construction. Under those conditions, very strong selection was necessary to favor genetic differentiation of construction propensity among demes. We put forth a constitutive theory for the evolution of adaptive habitat construction that unifies our model with previous verbal and quantitative models into a formal conceptual framework.

## Graphical abstract

Adaptive habitat construction is a process by which individuals alter their environment so as to increase their (inclusive) fitness. We show that the conditions for such adaptations are narrow such that most effects of organisms on their environment are non‐adaptive.

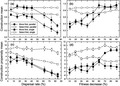

## 1 INTRODUCTION

All organisms, by their very existence, alter their environment as they take in and expel matter and energy. Very often those alterations affect the future fitness of those organisms and others around them. It is therefore unsurprising that natural selection would shape those alterations so as to increase the fitness of the organisms. If those alterations decrease the fitness of the organism, selection typically should act to minimize those effects. (We are ignoring selection for a beggar‐thy‐neighbor strategy, also known as spite (Hamilton, 1970)). In this paper, however, we explore the case where these alterations have positive effects. Such positive effects have been labeled “niche construction” (Odling‐Smee et al., 2003, 2013); we prefer the terminology of Sultan (2015)—“habitat construction”—as niche construction has been used to refer to a large variety of ways that an organism can alter its fitness, by changing its own phenotype, altering the surrounding environment, or by simply moving to an alternative environment.

Habitat (niche) construction activities have been claimed to be adaptations that have come about through natural selection (Odling‐Smee et al., 2003, 2013; Sultan, 2015). That claim can be examined based on the form of that construction and the set of individuals that the environmental alterations would affect. Obvious adaptations are the various examples of artifact construction (sensu Odling‐Smee et al., 2013). Such artifacts include bird nests, beehives, termite mounds, and beaver dams. Despite the costs to the individuals of such elaborate constructions, they also have obvious benefits to those individuals that result in a net increase in fitness.

Less obvious as adaptations are instances where individuals simply alter environmental conditions. One hallmark of artifact construction is that its benefits are nearly always directed at the constructing individual or its immediate kin. General alterations of the environment are typically undirected, affecting all individuals in the vicinity of the constructing individual, which may include effects on other species. For example, earthworms, as they burrow through the soil, alter its consistency (Darwin, 1892). Elephant browsing can create arboreal nesting sites for lizards (Pringle, 2008). Grass can stabilize dune systems, setting the stage for the establishment of other species (Cowles, 1899). Some species of chaparral vegetation may have evolved for increased flammability (Cowan & Ackerly, 2010; Schwilk, 2003), which in turn has multiple effects on the rest of the community (Montenegro et al., 2004; Pausas et al., 2017). Litter decomposition is a form of environmental conditioning that is potentially a co‐evolved relationship between plants and soil microbes. (See Post & Palkovacs, 2009; Sultan, 2015 for an extensive list of such types of alterations.) These types of environmental alterations are much more widespread than artifact construction. But are all such alterations adaptations, or are most simply non‐adaptive, incidental effects that are not directly selected for? In this study, to differentiate positively selected, adaptive habitat construction from non‐adaptive, incidental effects, we term the latter “environmental conditioning.” Because fitness benefits may extend to unrelated individuals, the conditions that select for such types of habitat construction may be more restricted.

The goal of this paper is to explore what factors might favor habitat construction when the benefits of environmental alterations are shared by many individuals of the same species. By determining those conditions, we can set bounds on the likelihood that such alterations are adaptive. Furthermore, the conditions favoring such undirected benefits for a single species are more favorable than those in which the benefits are shared by multiple species where co‐evolutionary dynamics weaken selection (Matessi & Jayakar, 1976; Trivers, 1971; Wilson, 1990). Thus, our results potentially put further restrictions on claims about niche construction as a general, adaptive condition.

### 1.1 Questions addressed and model predictions

We use individual‐based simulations to explore the factors that might affect selection for or against habitat construction. In our model, the environment exists in a baseline state. That state differs from the one that would result in maximal fitness of the individuals. Individuals can alter that environment – do construction – so as to move the environment toward that optimum. Conversely, the environment tends to decay back toward the baseline state. The entire population is divided into multiple demes. Although linked by dispersal, within a deme any alteration of the environment due to construction is independent of such alterations in other demes.

We address two broad themes. The first theme examines the factors that determine who receives the benefits of construction: the individuals doing the constructing, their immediate kin, or unrelated individuals. We do that by varying the size of demes, dispersal rates, the timing of dispersal relative to construction and selection, and the temporal sustainability of construction effects (i.e., the decay rate). These factors probe the strength of diffuse selection on groups of potentially related individuals, that is, kin selection. In the initial simulations, the environment was uniform and unstructured; both the baseline and optimal environments were the same in all demes and dispersal was equally likely between all demes.

Because construction is costly, it should be favored when the benefits of habitat construction are enjoyed either by the individuals that bore the costs or their close relatives (e.g., offspring); such benefit sharing is a form of inclusive fitness through group selection (Hamilton, 1964; Wilson, 1983). Based on previous models of the evolution of habitat construction (Chisholm et al., 2018; Krakauer et al., 2009; Kylafis & Loreau, 2008; Laland et al., 1996; Lehmann, 2008; Silver & Di Paolo, 2006), we expect that the propensity for construction should increase when the benefits are more likely to be directed to self or near kin in the current or immediately following generations: (1) as the number of individuals in a deme decreases, (2) as the decay rate increases, (3) as the dispersal rate decreases, and (4) when an individual does construction in the same deme as it or its offspring experience selection. We recognize that this first theme is primarily confirming previous results; however, such confirmation is necessary to demonstrate that our model is behaving as expected. Equally important, these initial simulations are necessary to determine and justify the parameter values used in our second theme.

The second theme examines how environmental heterogeneity and structure can affect that selection: temporal versus spatial heterogeneity, spatial variation in the optimal amount of construction, and the relative patterns of dispersal and spatial heterogeneity. These factors indicate how narrow or broad are the environmental conditions that will select for construction. In the simulations involving spatial heterogeneity, baseline environmental conditions differed among the demes in a structured way by the existence of an environmental gradient and, for some simulations, dispersal that was limited to demes that were adjacent or very close along that gradient. We predict that selection for construction should be weakened under three conditions: increasing temporal variation, when there is spatial variation in the optimal amount of construction, and when the pattern of dispersal does not match the spatial pattern of environmental heterogeneity.

## 2 THE MODEL

### 2.1 Model structure

The model was a discrete‐time, individual‐based simulation implemented in Fortran 77 that used a gene‐based model of adaptation. The variables and parameters are listed in Table 1. The genotype of an individual consisted of ten loci – two types of five each – that were unlinked within and among types: genes determining the phenotype (trait loci) and genes determining the amount of change in the habitat that an individual would make (construction loci).

**TABLE 1:** Variables and parameters for the model simulations

Symbol	Meaning	Value
(A) For all simulations		
*T*	Phenotype of an individual	
*G*	Trait allelic value	
*C*	Construction allelic value	
*A*	Construction propensity of an individual	
*B*	Amount of construction by an individual	
*θ*	Baseline environment in each deme	
*E*	Environment in each deme at the end of a generation	
*δ*	The rate of decay of the environment to the baseline	50%
Δ*H*	The total construction in a deme in a generation	
*S*	Environment in each deme at the time of selection	
*T_opt_*	Optimum phenotype in a deme	
*W*	Individual survival probability from juvenile to adult	
*i*	Subscript for *i*th deme	
*j*	Subscript for *j*th individual	
*k*	Subscript for *k*th allele	
*t*	Subscript for the *t*th generation	
	Number of trait loci	5
	Number of construction loci	5
*ω*	Strength of selection	4
*γ*	Cost of construction	0.002
	Per‐generation per‐locus mutation rate	0.1
	Variance of mutation effect	0.01
(B) For unstructured environment simulations		
*φ*	Average fitness decrease in the baseline environment	50%
	Dispersal rate	4%−100%
	Number of demes	256, 128, 64, 32,16
*N*	Number of individuals per deme after reproduction	2, 4, 8, 16, 32
*τ*	Amount of temporal variation scaled as a percent of the difference between the baseline and the optimum	0%−27.5%
(C) For structured environment simulations		
*φ*	Average fitness decrease in the baseline environment	50% or 10%−90%
	Dispersal rate	4%−100% or 41%
	Number of demes	50
*N*	Number of individuals per deme after reproduction	8

### 2.2 Determining the environment – structure

For simulations involving an unstructured, uniform environment, all demes consisted of the same baseline environment. Habitat construction (described below) increased the environmental value away from the baseline, and subsequent decay moved it back toward the baseline. The optimal environment was also the same for all demes and 10 units greater than the baseline environment. The number of demes was 16, 32, 64, 128, or 256, and the respective carrying capacity for each deme was 32, 16, 8, 4, or 2; thus, the total metapopulation size (512) was the same for all simulations.

**FIGURE 1:**
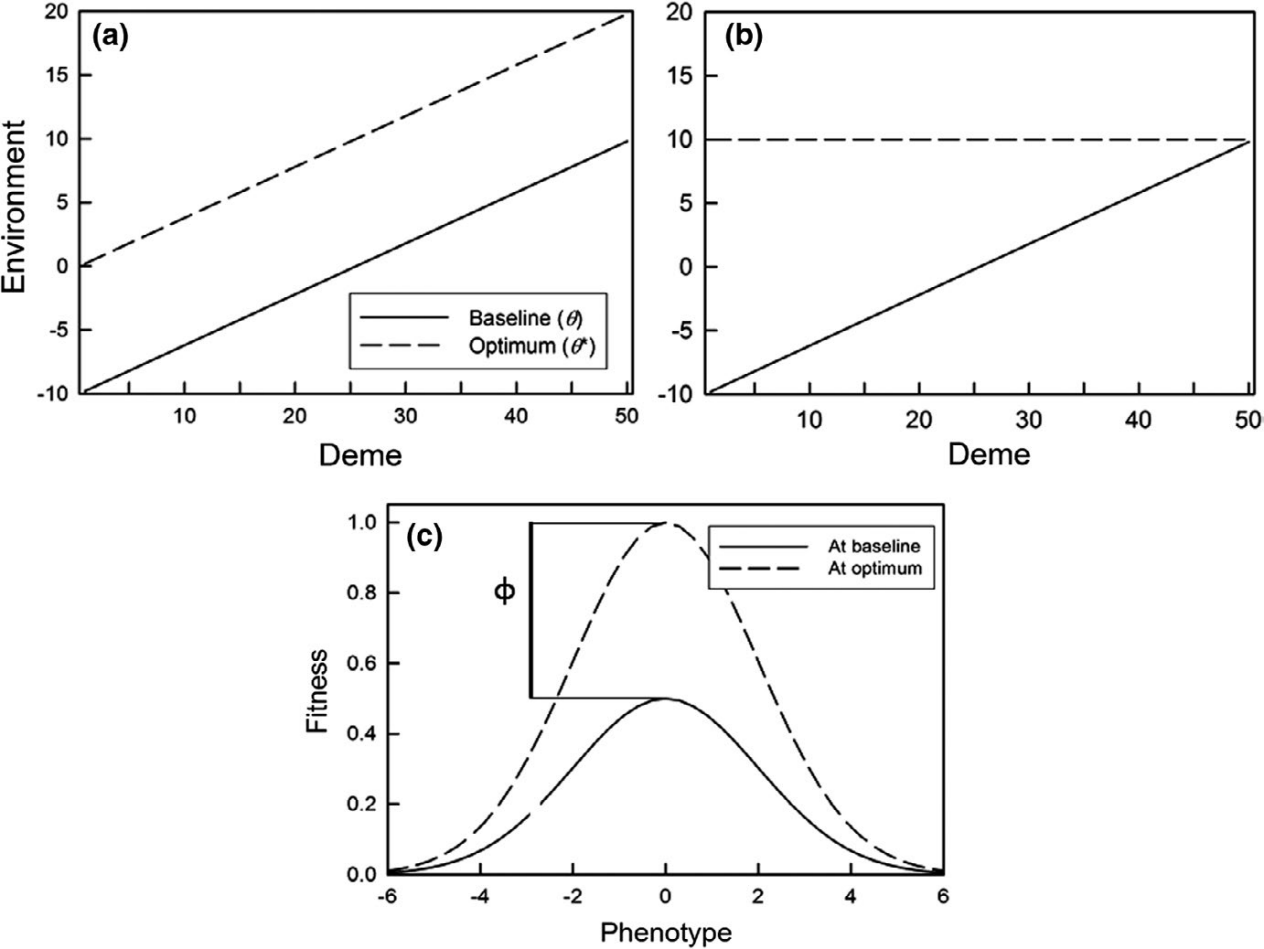
(a) Both the baseline (*θ*) and optimal (*θ* *) environments vary along a gradient (parallel optimum). (b) The baseline varies, but there is a single optimum for all demes (single optimum). (c) The fitness function in a given deme when the environment equals the optimum, and the decrease in fitness (*φ*) when the environment equals the baseline (for the single optimum case, this is the decrease for the middle of the gradient, which is also the average decrease across all demes), if the optimum phenotype is 0; shown is a value of *φ* = 50%. Trait values are in the same units as the environment

For simulations involving a structured gradient, the metapopulation consisted of a linear array of 50 demes (indexed by *i* from 1 to 50; Figure 1). The carrying capacity for each deme was 8, for a total metapopulation size of 400. A baseline environmental gradient (environment in the absence of construction; Figure 1a,b, solid lines) was created by varying the environmental value (*θ_i_*) in a linearly increasing fashion along the array from approximately −10 arbitrary units at one end of the gradient to about +10 units at the other; the environments in adjacent demes differed by 0.4 units [*θ_i_* = 0.4(*i* – 25.5)]. Each deme also had an optimal environment (*θ_i_* *) that was either 10 units above the baseline (Figure 1a; dashed line) or fixed at 10 units (Figure 1b; dashed line). The optimal amount of construction was, therefore, the same in all demes (Figure 1a) or varied among demes (Figure 1b). Again, habitat construction increased the environmental value away from the baseline, and subsequent decay moved it back toward the baseline.

### 2.3 Determining the environment – construction

Between generations, the environment in each deme (*i*) decayed back toward its baseline state. The decay between the end of generation *t* – 1 and the start of generation *t* (Δ*E_it_*) was

(1) $$\Delta E_{\mathrm{it}}=‐\delta \left(E_{i(t‐1)}‐\theta_{i}\right),$$

where *E_i_*_(_ *_t_*_−1)_ is the environment in deme *i* at the end of the generation *t *− 1 and *δ* is the rate of decay. This produced an environment of *E_i_*_(_ *_t_*_−1)_ + Δ*E_it_* before construction. For simulations that explored the effects of decay rate, *δ* varied from 10% to 100%; otherwise, *δ* was fixed at 50%.

**FIGURE 2:**
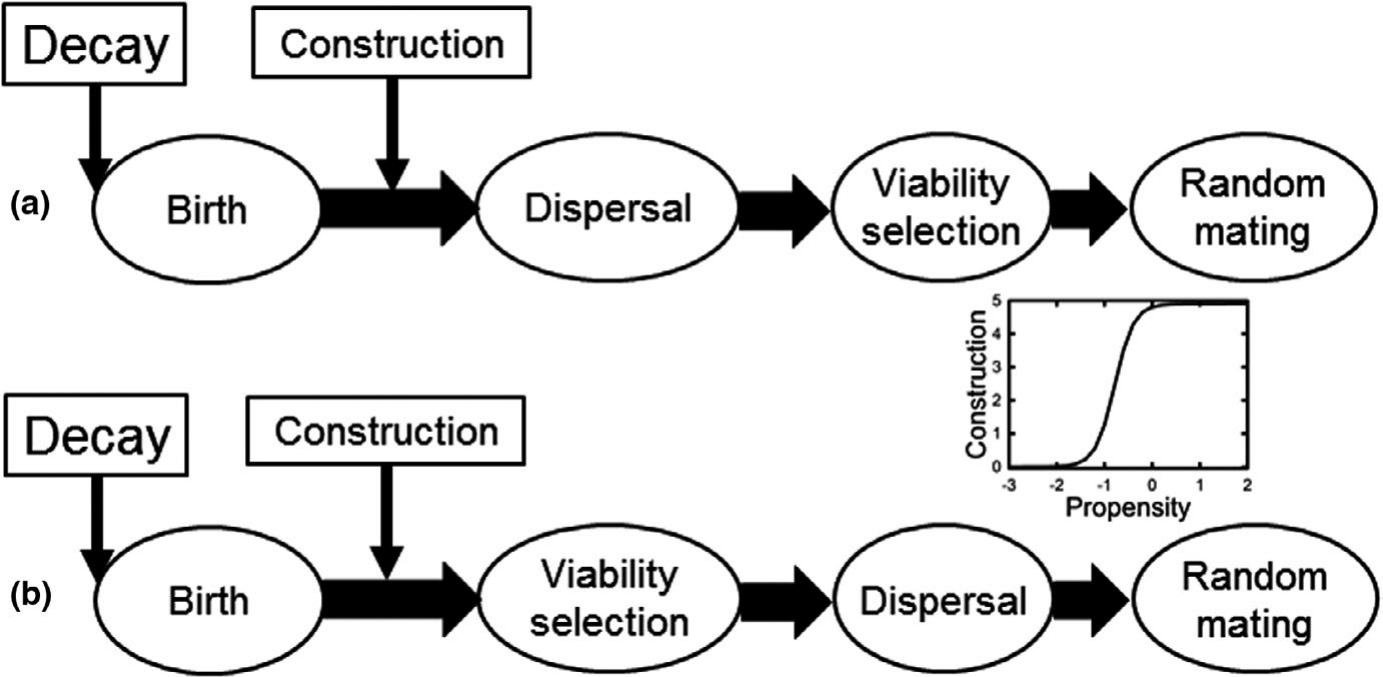
The two life history patterns that were modeled. (a) Birth, construction, dispersal, selection, reproduction, and death (“move first”). (b) Birth, construction, selection, dispersal, reproduction, and death (“select first”). Insert: The amount of construction by an individual as a function of its construction propensity (sum of the construction alleles)

Habitat construction occurred after birth prior to either dispersal or selection (Figure 2). The amount of habitat construction that occurred in each deme in each generation was determined by two functions: the amount of construction attempted by each individual (a function of its genotype) and the amount of construction by the entire deme (a function of the individual constructions). The construction propensity of an individual was the sum of five unlinked diploid construction loci:

(2) $$A_{ijt}=\sum_{k=1,10}^{}C_{ijkt}$$

where *C_ijkt_* is the allelic value of the *k*th construction allele of the *j*th individual in the *i*th deme in generation *t* and *A_ijt_* is that individual's construction propensity. The amount of construction (*B_ijt_*) by an individual was a logistic function of its construction propensity:

(3) $$B_{\mathrm{ijt}}=\frac{5}{\left[1+\exp(‐5A_{\mathrm{ijt}})\right]}$$

(Figure 2b inset). The construction in the *i*th deme in generation *t* (Δ*H_it_*) was a saturating function of the sum of the construction of all *N_i_* individuals in the deme:

(4) $$\Delta H_{\mathrm{it}}=\frac{\left(\sum_{j=1,N_{i}}B_{\mathrm{ijt}}\right)}{\left(1+0.2\sum_{j=1,N_{i}}B_{\mathrm{ijt}}\right)}.$$

The maximal amount of construction in a single generation was 5.0 units; the mean optimum environment was 10 units greater than the baseline environment (Figure 1a,b).

The environment in the *i*th deme at the time of selection was the environment at the end of the previous generation plus the changes due to decay and construction:

(5) $$S_{\mathrm{it}}=E_{i(t‐1)}+\Delta E_{\mathrm{it}}+\Delta H_{\mathrm{it}},$$

which was also the environment at the end of generation *t* (*E_it_*). (Our choice of specific parameter values here and below affects the quantitative details of our conclusions, but not the overall qualitative patterns.)

The form of habitat construction that we model is “unresponsive” as the amount of construction performed by an individual is based solely on its genotype. In contrast, “responsive” construction would entail an individual assessing the state of the environment first, then doing only that amount of construction necessary to reach the optimal state. We used a different form of restraint in our model; construction was limited due to the two saturating functions (Equations 3 and 4). For a deme as a whole, the total amount of construction was a saturating function, premised on there being some type of feedback among individuals limiting what any single individual could accomplish. For a single individual, the saturating function was premised on the notion that a single individual cannot perform an unlimited amount of construction due to energy, time, or other constraints. We emphasize that the results of our modeling are dependent on all of these choices. Models based on other types of habitat construction might reach different conclusions.

### 2.4 Determining the phenotype

An individual's phenotype (trait value) was determined at birth by five unlinked diploid trait loci. The loci contributed additively to the trait, which for simplicity was a scalar with units of equivalent magnitude as the environment: $T_{\mathrm{ijt}}=\sum_{k=1,10}\;G_{\mathrm{ijkt}}$, where *T_ijt_* is the phenotype of the *j*th individual that develops in the *i*th deme in generation *t*, and *G_ijkt_* is the value of the *k*th trait allele of that individual. There was no random component of an individual's phenotype.

### 2.5 Selection

Life history events occurred in one of two sequences (Figure 2): (1) birth (when the phenotype is determined), then dispersal, selection, and reproduction (denoted as “move first”); or alternatively, (2) birth, selection, dispersal, and then reproduction (denote as “select first”). All individuals die after reproduction. Selection occurred during survival from juvenile to adult. The survival probability of each individual was a Gaussian function of the difference between its phenotype and the optimum phenotype in deme *i* at time *t* (*T*_opt,_ *_it_*) (first term) minus the cost of construction (second term):

(6) $$W_{\mathrm{ijt}}=f_{\mathrm{it}}\cdot \exp\left\{‐\frac{1}{2}{\left(\frac{T_{\mathrm{ijt}}‐T_{\text{opt},it}}{\omega }\right)}^{2}\right\}‐\gamma B_{\mathrm{ijt}},$$

where *f* is a function (see below) that accounts for a decrease in fitness due to the difference between the current environment and the optimum environment (Figure 1c) and *ω* determines the strength of selection on the phenotype (a lower value being stronger selection). Because we set units of trait values to be of equivalent magnitude to environmental units, *T*_opt,_ *_it_* directly equals *S_it_* without need of a transformation. For all simulations, *ω* = 4. For the structured environment, the length of the spatial gradient across all demes was approximately 2.5 times the width of the within‐deme selection function (2*ω*). Habitat construction was costly; γ was the per‐unit construction cost, which was multiplied by the construction trait as defined in Equation (3). The cost parameter (γ) chosen was based on the percentage decrease in total fitness (survival probability) for individuals that expressed the optimum phenotype, so an individual that contributed the maximal construction would experience a 1% decrease in fitness. Although in the simulations this cost function allowed for the possibility of negative fitness values, such negative values simply meant that an individual had a 0% probability of survival.

For habitat construction to be selected for, construction has to increase fitness. That construction benefit was embodied in the *f* term in Equation (6), which was calculated as:

(7) $$f_{\mathrm{it}}=1‐\varphi \left|\frac{\theta_{i}^{*}‐S_{\mathrm{it}}}{\theta_{0}^{*}‐\theta_{0}}\right|.$$

For simulations with unstructured environments: *θ*_0_ * − *θ*_0_ = 10. For simulations with a gradient, *θ*_0_ * and *θ*_0_ are the optimal and baseline environments at the center of the gradient (between demes 25 and 26); the difference (denominator) also equals 10 for these simulations. This function (*f_it_*) equals 1.0 when the environment in the *i*th deme at the time of selection (*S_it_*) equals the optimum environment in that deme (*θ_i_* *), falls linearly with the absolute value of the difference between *S_it_* and *θ_i_* *, and reaches a minimum of 1 − φ when *S_it_* is at the baseline (*θ_i_*) in the center of the gradient (Figure 1c). Selection on environmental construction is therefore toward the optimum, and the greater the value of φ, the greater the strength of selection on that construction. For the parallel optimum, this reduction in fitness at the baseline is the same in all demes. For the single optimum, this reduction is highest on the left of the gradient and lowest on the right, with φ being the average across all demes. The total change in the environment due to construction was not limited (except by the maximum per‐generation construction and the decay rate); it could increase the selective environment (*S_it_*) to be greater than the optimum (*θ_i_* *), which would cause a similar reduction in fitness.

### 2.6 Temporal variation

In some simulations with the unstructured environment, there was also random variation added to one of two aspects of that environment, either the selective environment experienced by individuals (*S_it_*), or the optimal environment (*θ_i_* *). That variation also had two spatial patterns: either it was independent among demes, that is, each deme experienced a different pattern of variation, or all demes experienced the same pattern of variation. Variation in the environment was added at the time of construction. Random variation within each deme was simulated as a sequence of independent zero‐mean Gaussian random deviates (*z_it_*) with a standard deviation of *τ* that was scaled to be a percentage of the initial difference between the baseline and optimal environments (10 units for these simulations). If each deme experienced a different pattern of variation, the environment in the *i*th deme at the time of selection was as follows:

(8) $$S_{\mathrm{it}}=E_{i(t‐1)}+\Delta E_{\mathrm{it}}+\Delta H_{\mathrm{it}}+z_{\mathrm{it}}$$

(compared with Equation 5). If all demes experienced the same pattern of variation, *z_it_* was replaced with *z_t_*, that is, the same deviation in each deme. Variation in the optimal environment occurred at the time of selection, using similar Gaussian random deviates (*θ_it_* * = *θ_i_* * + *z_it_* or *θ_it_* * = *θ_i_* * + *z_t_*), depending on whether that variation was independent or correlated among demes. Regardless of the existence of any extrinsically imposed temporal variation, the dynamic of construction and decay always produced autocorrelated temporal variation within each deme.

### 2.7 Environmental structure and dispersal

Dispersal occurred in one of two patterns that corresponded to the two types of environments: island for the unstructured environment and stepping‐stone for the structured environment. For the island pattern of dispersal, if an individual moved it had an equal probability of moving to any of the other demes. (In ecology, this pattern is referred to as an unstructured metapopulation dispersal pattern.) The propensity to disperse was fixed (non‐evolving), dispersal probabilities were identical for all individuals, and dispersal per se had no cost – survival during dispersal was 100%.

For the stepping‐stone pattern of dispersal, the dispersal probability was determined using a zero‐mean Gaussian random number, which in turn determined the number of demes through which an individual moved; the integer part of the random number determined the number of demes moved and the sign determined the direction of movement (see figure 1 of Scheiner & Holt, 2012). The result was that the probability of moving and the average number of demes moved were correlated, with most individuals that moved only moving one deme and the rest moving at most a few demes. Individuals who would have moved beyond either end of the gradient stopped at the end deme. Again, the propensity to disperse was fixed (non‐evolving), dispersal probabilities were identical for all individuals, and dispersal per se had no cost.

For simulations that explored the effects of dispersal, the dispersal rate varied from 4% to 100%. Otherwise, the dispersal rate was fixed at 44% for unstructured environments or 41% for structured environments; these values were chosen based on the results of the simulations that examined the effects of dispersal rate.

### 2.8 Reproduction and mutation

Sexual reproduction by surviving individuals was accomplished by assembling pairs of individuals within a deme at random with replacement (allowing for self‐fertilization), with each parent producing a haploid gamete of unlinked alleles. Each pair then produced one offspring. This process was repeated until the carrying capacity of that deme was reached. This procedure assumes soft selection within each deme because population size (after reproduction) was determined independently of the outcome of selection; because individuals within a deme compete to produce successful offspring, such a procedure will weakly oppose kin selection by increasing kin competition when the deme size is very small (Wade, 1985). The model assumes that the spatial scale of reproduction and mating matches that of density dependence and the grain of the selective environment.

When new offspring were generated, each allele at each locus mutated with a probability of 10%. In general, lower mutation rates simply lengthen the time scale over which evolution happens without affecting the eventual outcome, for the kinds of models considered here (Scheiner & Holt, 2012). In addition, this somewhat high mutation rate has the virtue of minimizing linkage disequilibrium. When a mutation occurred, the allelic value was changed by adding a Gaussian deviate (mean of 0 and a standard deviation of 0.1 units) to the previous allelic value (i.e., this is a continuum‐of‐alleles model, Kimura, 1965). Allelic values were unconstrained. Trait alleles – and the subsequent phenotypes – could take any value from −∞ to ∞. Similarly, the construction alleles could take any value from −∞ to ∞.

### 2.9 Initial conditions

Each simulation was initialized with individuals newly born in each deme at that deme's carrying capacity. For each individual in the initial generation, allelic values for the trait and construction loci were chosen independently from the values −2, −1, 0, 1, and 2, with each value being equally likely. Even though these alleles were integer‐valued initially, their values could assume any real number in subsequent generations due to mutation. The environment of each deme was initially equal to its baseline. The initial expected value of construction propensity was 0, so that the initial expected value of potential construction (*B_ij_*_0_) of each individual was equal to 2.5 (Figure 2, inset). There was, therefore, a significant amount of construction in early generations. Such construction lessened the probability of immediate extinction in simulations with large values of *φ*.

### 2.10 Response variables

All simulations were run for 1000 generations to ensure that equilibrium (the point after which all calculated quantities showed no further obvious directional trend) was reached. Each parameter combination was replicated 20 times; the results shown are the means and standard errors of those replicates.

To assess evolutionary outcomes, at the end of 1000 generations there was one last round of mating and reproduction (without environmental decay) to return the demes to full size before parameters were calculated. For unstructured environments, evolutionary outcomes were assessed by examining the mean of the total construction within demes (*E_it_*), the mean of the construction propensity of individuals (*A_it_*), and the mean fitness (*W_tt_*). The parameters were measured by first averaging among individuals within demes, and then averaging among demes. For structured environments, for each of these parameters we also examined the slope along the gradient. Slopes were calculated as a linear regression on the deme averages. For construction propensity, the slope was a measure of genetic differentiation among demes. All slopes were standardized relative to the slope of the baseline environment (Figure 1a,b).

For total construction, the environmental values (*E_it_*) were averaged across all demes. This average was divided by 10, so a value of 1.0 indicates that habitat construction moved the average environment to match the optimum at the midpoint of the environmental gradient, which was always 10 units higher than the baseline; no construction would result in a value of 0. (The amount of construction in deme *i* is actually *E_it_* – *θ_i_*, but since the average *θ_i_* is 0, when this is averaged across demes, it is equal to the average *E_it_*). For the parallel optimum, a slope of 1 indicates that habitat construction resulted in an environment that matched the slope of the optimal environment across the gradient; for the single optimum, a slope of 0 indicates that habitat construction caused the environment to match the slope of the optimal environment across the gradient.

## 3 RESULTS

### 3.1 Unstructured environments and no environmental heterogeneity

We predicted that habitat construction would be favored when the benefits of construction are more likely to flow to the individual doing the constructing or its close kin. We tested that prediction by manipulating the rate at which the constructed environment reverted to its baseline state, the deme size, and the rate of dispersal. For the “select first” life history pattern, the individual doing the construction always directly benefits from the construction. For the “move first” life history pattern the constructor benefits only if it does not disperse. For both patterns, a lower dispersal rate results in more of the benefits accruing to the constructor's descendants. Similarly, the faster the environment reverts to the baseline state, the more the benefits of construction are focused on one's direct offspring. We found that all of these factors interacted.

**FIGURE 3:**
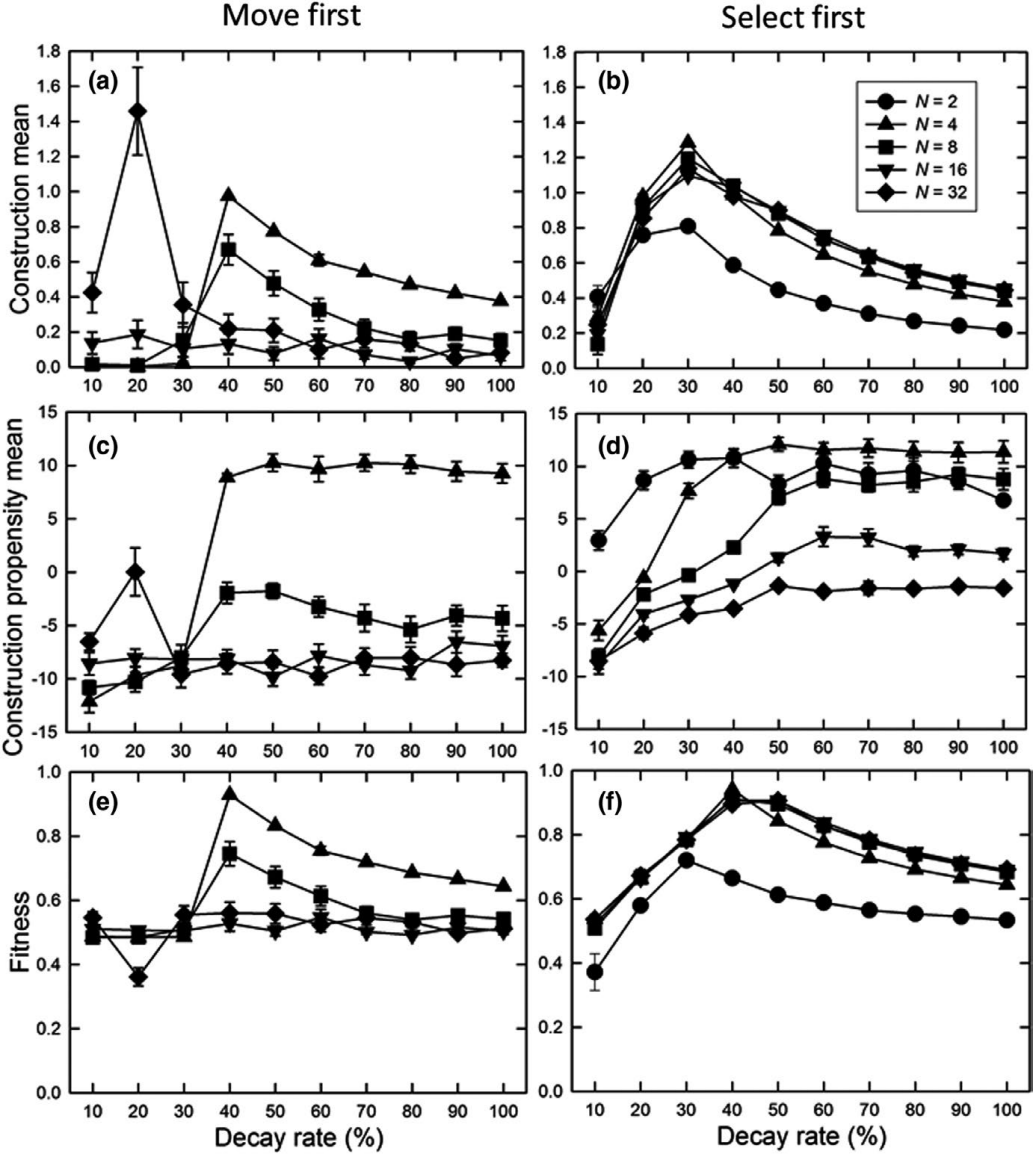
For the unstructured, uniform environment, the effect of the decay rate (*δ*) on (a, b) the normalized construction environmental mean (mean *E_it_*/10), (c, d) the mean construction propensity of individuals (mean *A_ijt_*), and (e, f) final mean fitness (*W_ijt_*) for the (a, c, e) “move first” and (b, d, f) “select first” life history patterns, for different population sizes. Dispersal was the island pattern; the dispersal rate was 44%; and the total number of individuals in the metapopulation was 512 for all population sizes. Shown are means and standard errors of 20 replicates; when error bars are absent, they are smaller than the symbol. If values are missing, those parameter combinations resulted in extinction of the metapopulation in all of 60 replications

Our first set of simulations examined an unstructured environment (i.e., the baseline and optimal environments were the same in all demes) with a matching pattern of dispersal (i.e., an island pattern where all demes were equally distant). If dispersal occurred before selection (“move first”), the greatest propensity for construction (average construction phenotype, mean *A_ijt_*) occurred for small population sizes (*N* = 4) at fast rates of decay (Figure 3c), with a maximum amount of construction (mean *E_it_*/10) at a decay rate of 40%. (For *N* = 2, the metapopulation simply went extinct under these parameter values.) For intermediate‐to‐high decay rates, fitness declined with increasing population size for the smaller deme sizes (4, 8; Figure 3e).

For the largest population size (*N* = 32), there was almost no construction (mean *E_it_*/10) at high decay rates, with a peak at 20% (Figure 3a). That peak was due to a bimodal distribution of environmental construction values with about half near zero and about a third near the maximum possible value (2.4; not shown), suggesting the existence of alternative (quasi‐) stable states. The reason for this bimodality is that sometimes the metapopulation is getting “trapped” in an excessive construction phenotype. Because the life history pattern is “move first” and the dispersal rate is 44%, there is a (partial) disconnect between how much construction an individual does and its fitness outcome. In this case, the decay rate is slow enough that once the population is above the optimum it never moves the environment across that optimum threshold back to the state where no construction is selected for. While this result is relevant to only a narrow parameter range in our particular model, it may point at an interesting biological scenario that might possibly be more widespread in other models; analytic treatment of this combination of conditions may be warranted.

In contrast, if dispersal occurred after selection (“select first”), the amount of construction (Figure 3b) and the subsequent fitness (Figure 3f) were similar for all but the smallest population size, and was highest at an intermediate decay rate. As predicted, the propensity for construction increased with the decay rate at all population sizes (Figure 3d). Assuming that a steady state is reached, construction must balance decay. Since the maximum construction per generation is 5, and the amount of construction is standardized by dividing by 10, the maximum relative construction is equal to 0.5/*δ*. For larger populations, the decrease in construction with increasing decay rate (for intermediate‐to‐high decay rates; Figure 3b) matched the expected equilibrium pattern. Small population sizes will have a smaller limit; for example, for *N* = 2, the maximum construction is 0.33/*δ*. Thus, construction was not favored at low decay rates because the benefits did not accrue to close kin, while at high decay rates construction was not favored because it was costly while not being able to maintain the demes at the optimum.

**FIGURE 4:**
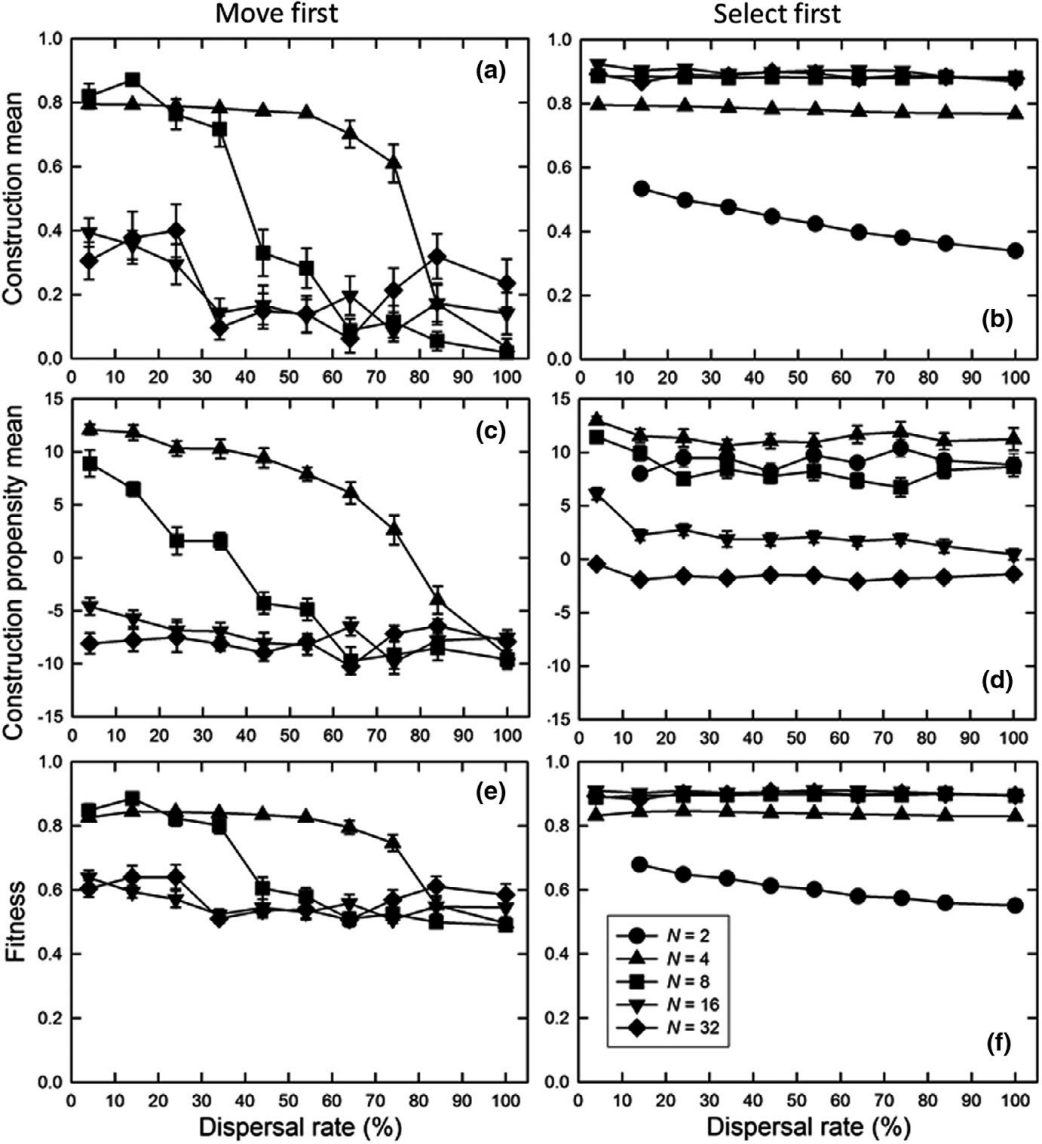
For the unstructured, uniform environment, the effect of the dispersal rate on (a, b) the normalized construction environmental mean (mean *E_it_*/10), (c, d) the mean construction propensity of individuals (mean *A_ijt_*), and (e, f) final mean fitness (*W_ijt_*) for the (a, c, e) “move first” and (b, d, f) “select first” life history patterns, for different population sizes. Dispersal was the island pattern; the decay rate (*δ*) was 50%; and the total number of individuals in the metapopulation was 512 for all population sizes. Shown are means and standard errors of 20 replicates; when error bars are absent they are smaller than the symbol. If values are missing, those parameter combinations resulted in extinction of the metapopulation in all of 60 replications

Dispersal rates had the predicted effect on construction for the “move first” life history pattern, with less construction as rates increased, especially at smaller population sizes (Figure 4a,c,e). (Again, the smallest population size resulted in metapopulation extinction.) In contrast, for the “select first” life history pattern, dispersal rate had no effect on construction, its propensity, or fitness, except for the smallest population size (Figure 4b,d,f). That is, because the constructing individual directly benefited, benefits to other individuals did not change the outcome. Overall, construction was favored the most when it benefits the conditioning individual or its immediate kin.

These results were used to set the parameters for the next sets of simulation that explored the effects of environmental structure and patterns of heterogeneity.

### 3.2 Unstructured environments and temporal heterogeneity

**FIGURE 5:**
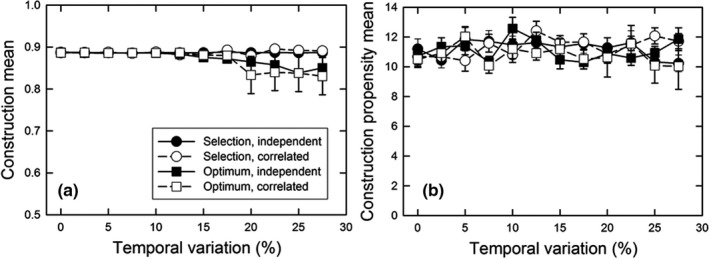
For the unstructured, uniform environment, the effect of different amounts of temporal variation on (a) the normalized construction environmental mean (mean *E_it_*/10) and (b) the mean construction propensity of individuals (mean *A_ijt_*). The variation could occur in the environment of selection (*S_it_*, circles) or in the optimum environment (*θ_i_* *, squares), and either vary independently (solid) or be correlated (open) among the demes. The standard deviation of temporal variation (*τ*) was scaled as a percentage of the difference between the baseline (*θ_i_*) and optimum environments (*θ_i_* *). The life history pattern was “select first”; dispersal was the island pattern with a rate of 4%. The population size (*N*) was 8, the number of demes was 64, and the decay rate (*δ*) was 50%. Shown are means and standard errors of 20 replicates; when error bars are absent, they are smaller than the symbol

For the unstructured environment, we looked at the effects of random environmental variation, using parameters that resulted in construction at or close to the optimal amount in the absence of temporal variation (see Figures 3 and 4). We examined temporal variation in either the environment of selection or in the optimal environment. That variation was either independent in each deme or the same across all demes. We had predicted that less construction would evolve with increasing temporal variation. In contrast, we found that for all of those scenarios, there were no effects on the amount or propensity for construction, except for a slightly smaller amount of construction at high rates of temporal variation when it was correlated among demes (Figure 5).

### 3.3 Structured environments and spatial heterogeneity

**FIGURE 6:**
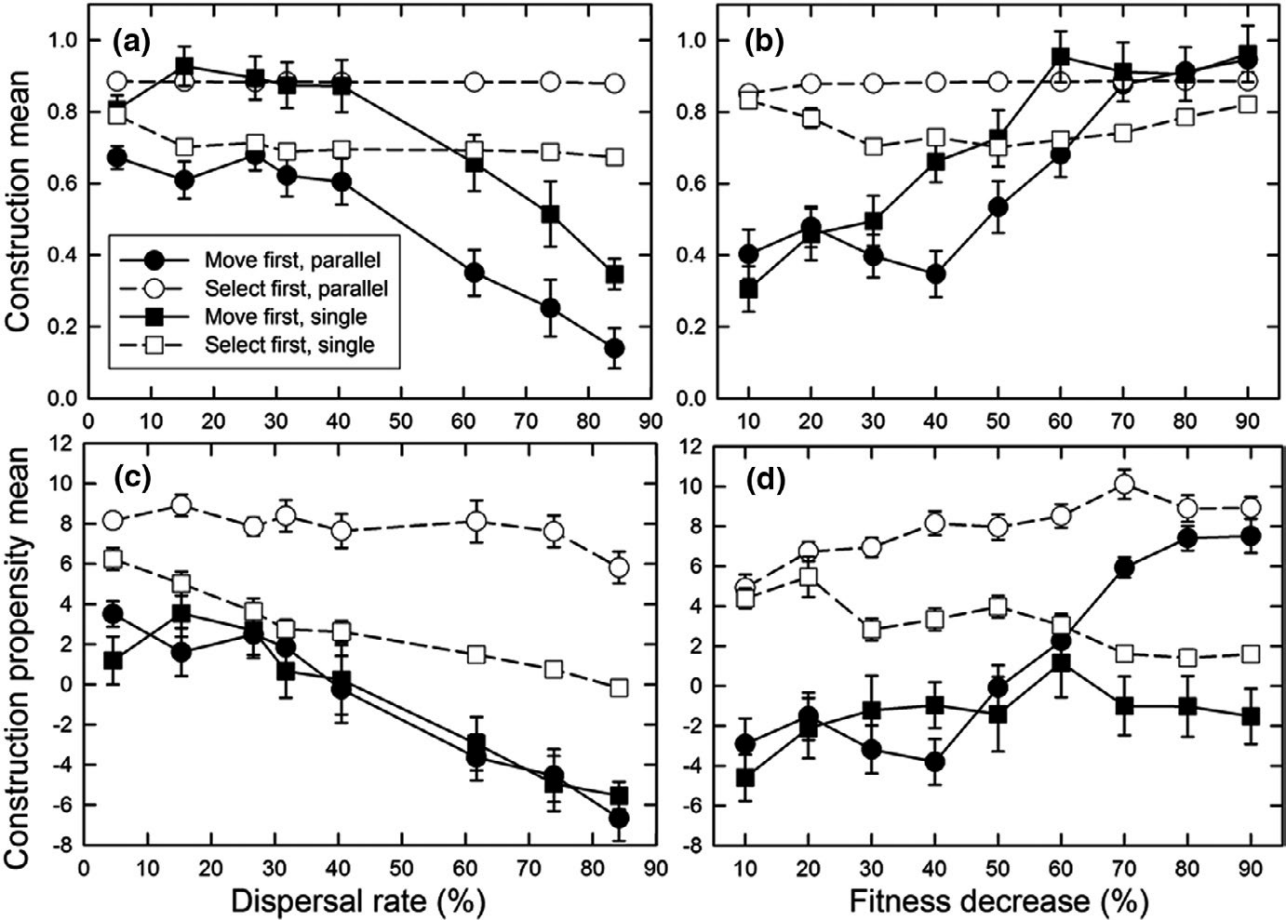
For the structured gradient environment, the effect of the dispersal rate (a, c) and the fitness decrease (*φ*) (b, d) on (a, b) the normalized construction environmental mean (mean *E_it_*/10), and (c, d) the mean construction propensity of individuals (mean *A_ijt_*) for both the patterns of environmental heterogeneity and life history orderings. The population size (*N*) was 8 and the decay rate (*δ*) was 50%. For (a, c), the fitness decrease (*φ*) was 50%; for (b, d), the dispersal rate was 41%. Dispersal was the stepping‐stone pattern. Shown are means and standard errors of 20 replicates; when error bars are absent, they are smaller than the symbol

We considered two types of structured environments, one in which the optimal amount of construction was the same in all demes along the environmental gradient (parallel optimum) and one in which the optimal amount decreased along the gradient (single optimum). In these simulations, the pattern of dispersal was a stepping‐stone, thus matching the gradient pattern of heterogeneity in the baseline environment. As with unstructured environments, a structured gradient generally favors construction at lower dispersal rates and when selection happens before dispersal (Figure 6a,c). These effects of dispersal timing could be offset, however, if the fitness benefits of construction were great enough (Figure 6b,d).

**FIGURE 7:**
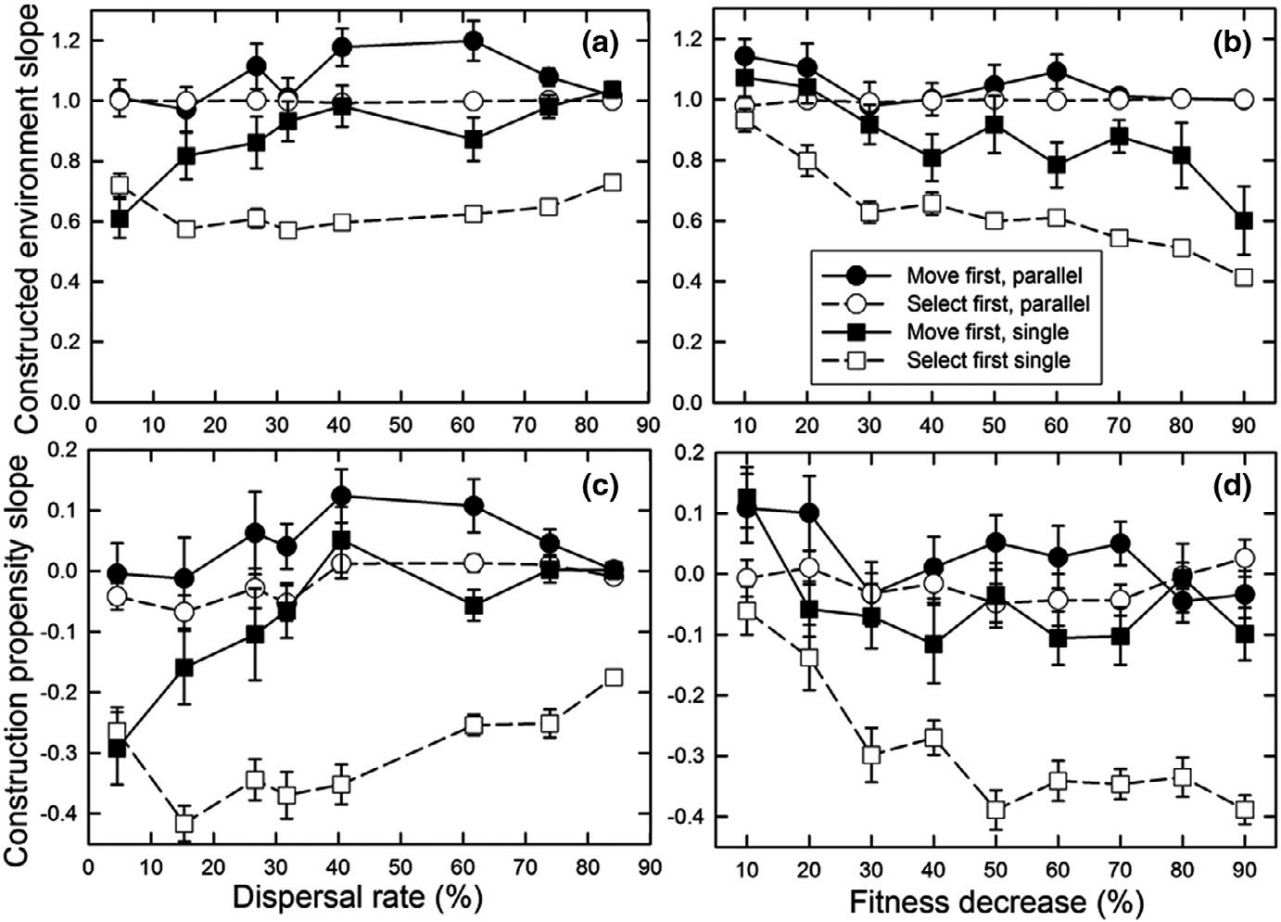
For the structured gradient environment, the effect of the dispersal rate (a, c) and the fitness decrease (*φ*) (b, d) on (a, b) the normalized slope of the constructed environment (slope *E_it_*/0.4), and (c, d) the normalized construction propensity slope (slope *A_ijt_*/0.4) for both the patterns of environmental heterogeneity and life history orderings. The population size (*N*) was 8 and the decay rate (*δ*) was 50%. For (a, c), the fitness decrease (*φ*) was 50%; for (b, d), the dispersal rate was 41%. Dispersal was the stepping‐stone pattern. Shown are means and standard errors of 20 replicates; when error bars are absent, they are smaller than the symbol

**FIGURE 8:**
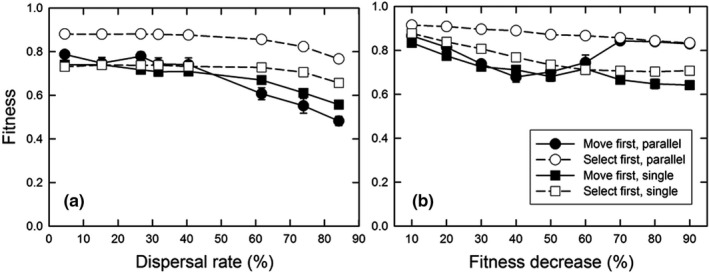
For the structured gradient environment, the effect of (a) the dispersal rate and (b) the fitness decrease (*φ*) on final mean fitness (*W_ijt_*) for both the patterns of environmental heterogeneity and life history orderings. For (a), the fitness decrease (*φ*) was 50%, and for (b), the dispersal rate was 41%. Dispersal was the stepping‐stone pattern. Shown are means and standard errors of 20 replicates; when error bars are absent, they are smaller than the symbol

The difference between the parallel optimum and single optimum scenarios is that the latter requires genetic differentiation in the amount of construction undertaken along the gradient to achieve maximum fitness, with the greatest amount of construction at the left‐hand end of the gradient and little to no construction at the right‐hand end (Figure 1b). For the parallel optimum scenario, perfect adaptation would entail a construction slope (normalized by the gradient slope) of 1.0 and a propensity slope of 0.0; for the single optimum scenario, the equivalent values would be 0.0 and −0.5. Values at or close to these ideals occurred only for the “select first” dispersal pattern and parallel optimum scenario (Figure 7). For the “select first” dispersal pattern and single optimum scenario, the construction slope was decreased, but not to zero; the dispersal rate had little effect on this slope (Figure 7a), but it declined as the fitness benefit of construction increased (Figure 7b). Genetic differentiation for construction propensity was greatest at lower dispersal rates (except the lowest; Figure 7c) and for greater fitness benefits (Figure 7d). For the “move first” dispersal pattern, such differentiation occurred only at the lowest dispersal rates (for the single optimum, of course). As a result, the highest fitnesses were seen for the “select first” dispersal pattern and parallel optimum scenario (Figure 8). In contrast, for the “move first” dispersal pattern, the two scenarios had similar fitnesses under nearly all conditions, except at very high fitness benefits where the parallel optimum resulted in fitnesses almost identical to those of “select first.”

### 3.4 Structured environments and non‐structured dispersal

**FIGURE 9:**
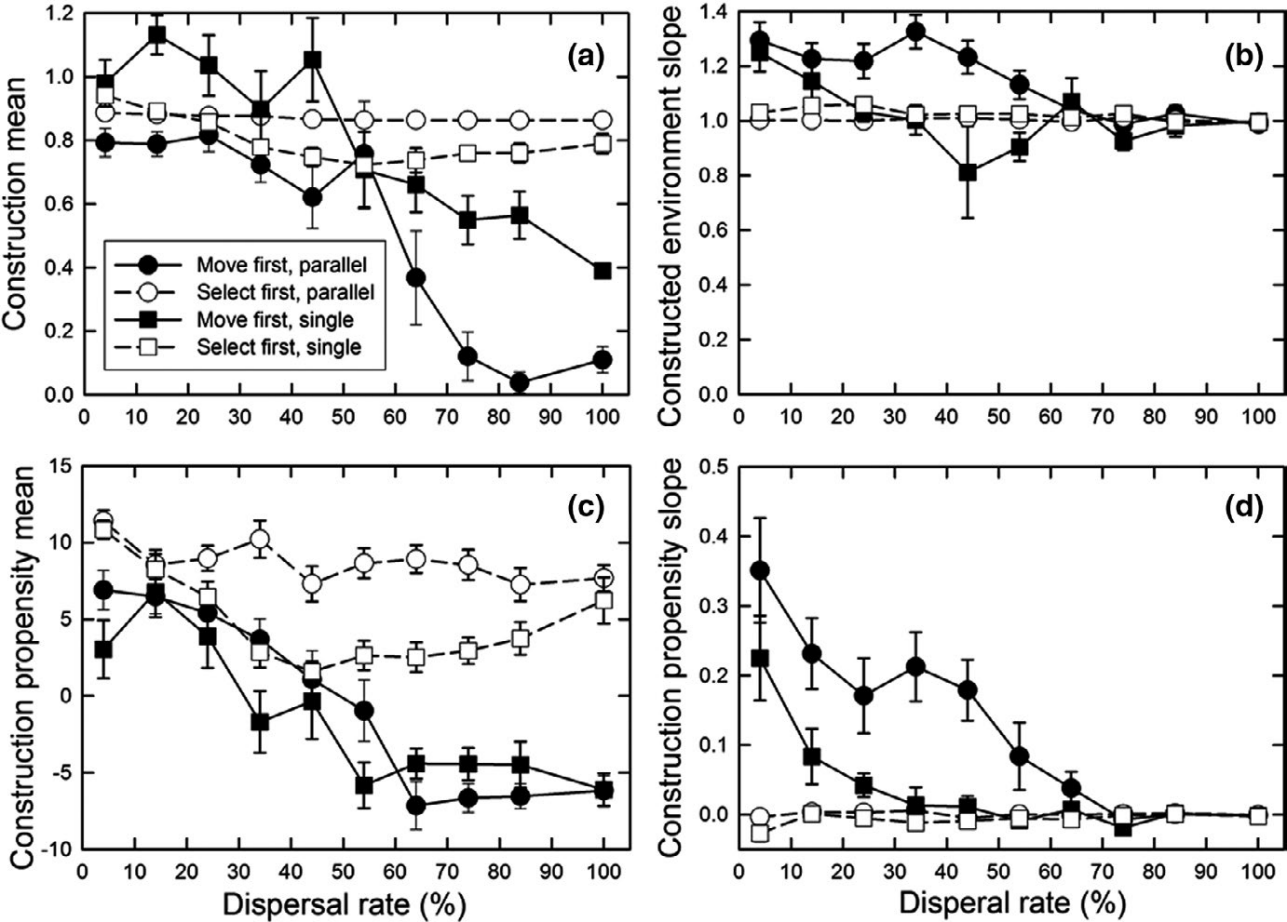
For the structured gradient environment with island‐type dispersal, the effect of the dispersal rate on (a) the normalized construction environmental mean (mean *E_it_*/10) (b) and the normalized relative slope of the constructed environment (slope *E_it_*/0.4), (c) the mean construction propensity of individuals (mean *A_ijt_*), and (d) the normalized construction propensity slope (slope *A_ijt_*/0.4) (c, d) for both the patterns of environmental heterogeneity and life history orderings. The fitness decrease (*φ*) was 50%. Shown are means and standard errors of 20 replicates; when error bars are absent they are smaller than the symbol

**FIGURE 10:**
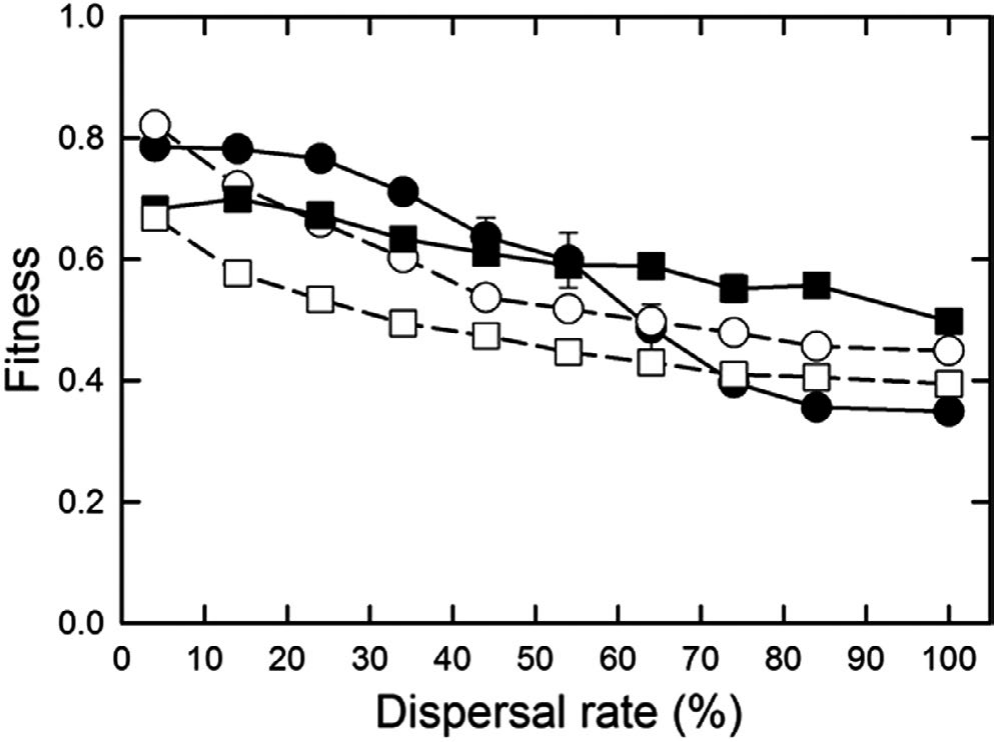
For the structured gradient environment with island‐type dispersal, the effect of the dispersal rate on final mean fitness (*W_ijt_*) for both the patterns of environmental heterogeneity and life history orderings. The fitness decrease (*φ*) was 50%. Shown are means and standard errors of 20 replicates; when error bars are absent, they are smaller than the symbol

Dispersal pattern and environmental structure can reinforce or oppose each other. A stepping‐stone dispersal pattern reinforces a structured environmental gradient in that individuals that move are most likely to land in an environment very similar to the one departed from. In contrast, an island (non‐structured) dispersal pattern matches an unstructured environment because traveling a greater distance does not result in traversing a greater environmental space. We tested the effects of a mismatch in these factors by exploring the effects of dispersal rates of an island dispersal pattern in a structured environment. Overall, the level of adaptation was less than for the stepping‐stone dispersal pattern (compare Figures 9a,b,c,d with 6a,c, and 7a,c, respectively, and Figure 10 with Figure 8a).

For the single optimum scenario, genetic differentiation for construction propensity failed to occur for the “select first” dispersal pattern (Figure 9d). Instead, the constructed environmental had a mean value that was slightly lower than the optimal mean (Figure 9a) and a slope that matched the baseline slope (1.0, Figure 9b).

For the “move first” dispersal pattern, at high dispersal rates there was selection for little or no construction, especially for the parallel optimum (Figure 9a,c). At low dispersal rates, the slope of the constructed environment was positive (Figure 9b), as was the slope of the construction propensity (Figure 9d) for both optimum patterns, but especially for the parallel optimum. These results mean that there was selection for more construction at the right‐hand end of the gradient (Figure 1a,b).

## 4 DISCUSSION

### 4.1 Theme 1: The recipients of construction benefits

Our results confirm the predictions of inclusive fitness and group selection theory (Hamilton, 1964; Wilson, 1983) and expectations based on previous models (Chisholm et al., 2018; Krakauer et al., 2009; Kylafis & Loreau, 2008; Laland et al., 1996; Lehmann, 2008; Silver & Di Paolo, 2006): construction will be more favored when its benefits are more likely to be directed to self or near kin (Figures 3 and 4). Unlike previous models, ours is an individual‐based simulation in which both the construction and trait phenotypes are multilocus. The general concordance of our results with those of the previous models suggests that these broad conclusions are robust.

The results of our model and others are in general accord with the examples of habitat construction that are typically touted. Examples of habitat construction described in Odling‐Smee et al. (2003) include: nests of cooperatively breeding birds, middens of woodrats, and burrows of mole rats. In such cases of artifact construction, the benefits of habitat construction are likely realized mainly by the constructing individual or its’ near kin. That is not to say that habitat construction cannot also benefit other individuals of the same or different species through environmental conditioning. What needs to be established is the extent to which such additional benefits are sufficiently strong and consistent to affect the evolution of those other individuals or to feedback on the evolution of construction (Odling‐Smee et al., 2003, pp. 298–301).

### 4.2 Theme 2: Environmental heterogeneity and structure

Our modeling efforts differ from previous ones by also exploring the effects of spatial and temporal variation. While two previous models included spatial structure (Lehmann, 2008; Silver & Di Paolo, 2006), environmental heterogeneity was generated only by the construction itself, as in our models under the first theme. In our simulations, contrary to our prediction, temporal variation had little to no effect on the evolution of construction (Figure 5). This lack of effects was likely because we focused on just the final equilibrium, and temporal variation acted as just background noise. Examination of the dynamics during early generations might show some effects.

Spatial heterogeneity did affect construction evolution, but those effects depended on various factors. As before, construction was favored when the timing and rate of dispersal relative to construction and selection resulted in the benefits of construction going to self or near kin. Conversely, construction was disfavored when the spatial pattern of movement did not match the spatial pattern of environmental heterogeneity (Figure 9). Notably, construction was less favored when there was spatial heterogeneity in the optimal amount of construction, especially as dispersal rates increased (Figures 6c and 7c). Very strong selection was necessary to favor genetic differentiation of construction propensity among demes (Figure 7d). This lack of differentiation contrasted with genetic differentiation for the trait itself, which always matched the constructed environment regardless of the pattern or rate of dispersal (results not shown). The reason that similar genetic differentiation did not occur for the propensity for construction is that selection on that trait is indirect, a process analogous to selection on modifier loci, which Wright (1934) showed to be weaker.

Because spatial heterogeneity is ubiquitous, the extent to which a lineage experiences that heterogeneity is a function of the rate of dispersal among locations and the distance of that dispersal relative to the grain of the environment. We predict that habitat construction will be greater when dispersal is limited. Because limited dispersal also tends to increase relatedness within demes, a test of this prediction will need to compare multiple populations that vary independently in dispersal rate and population size.

### 4.3 Habitat construction or environmental conditioning?

Our simulations suggest that adaptive habitat construction will be favored under a relatively narrow set of circumstances. First, the benefits need to be directed at oneself or near kin. Our model had a relatively small cost of construction. Increasing that cost should only further strengthen this requirement. It is notable that the most obvious examples of habitat construction are the creation of artifacts that very clearly fit this stricture: bird nests, beehives, termite mounds, and beaver dams. That is not to say that termite mounds and beaver dams do not also affect the environments of other species, but most likely those diffuse effects are ancillary and not the result of natural selection for them through those effects (contra Odling‐Smee et al., 2003, pp. 298–301).

Second, the pattern of environmental heterogeneity has to be conducive. Spatial variation in the optimal amount of construction appears to be an impediment to its adaptive evolution (assuming dispersal between locations), for the type of unresponsive construction explored here. For a discussion of responsive versus unresponsive construction in our models, see Scheiner et al. (2022). Given the ubiquity of environmental heterogeneity, these results suggest that selection on habitat construction may be constrained to reflect the average conditions in a landscape, rather than producing fine‐tuned results. This prediction can be tested by looking for genetic differentiation in the propensity for habitat construction. Additionally, the pattern of dispersal needs to conform to the pattern of spatial heterogeneity. That is to say, the indirect nature of selection on construction magnifies the known factors that limit adaptive evolution. More simulation work that delves deeper into those limitations is warranted.

On the other hand, habitat construction can create a positive feedback that maintains itself. Once construction exists and trait values evolve to that constructed optimum, joint selection on the trait and the propensity for construction will reinforce each other. Kylafis and Loreau (2008), using a scenario similar to ours, found two equilibrium points for construction, an unstable boundary point and a stable interior point. In our model, populations were initialized with a substantial amount of construction. These initial conditions were thus biased toward that stable interior point and might explain the bimodal result found for one parameter combination (Figure 3). A positive feedback can also be created between the amount of construction and environmental dynamics; for example, beaver dams can continue to accumulate naturally created woody debris. Such a positive feedback, by maintaining the constructed environment, might stabilize the equilibrium of the trait and construction propensity. On the other hand, if habitat construction is generally favored, if and only if it benefits the constructing individual or its immediate kin, then construction that benefits other species beyond tight mutualisms (i.e., community‐level selection) will be too diffuse to be selected for. Thus, a better understanding of the evolution of adaptive habitat construction awaits more detailed models combined with empirical data.

### 4.4 A constitutive theory of the evolution of habitat construction

A constitutive theory is a set of propositions that serve as guidelines or rules for building models within a defined domain (Scheiner, 2010; Scheiner & Mindell, 2019; Scheiner & Willig, 2011). They can unify a set of seemingly contradictory models (Leibold, 2011; Scheiner & Willig, 2005), crystalize a field around a theory (Fox & Scheiner, 2019; Gillespie et al., 2020), make explicit the sometimes tacit assumptions behind a model, reveal unexplored models (Fox et al., 2011), and help the conversion of a verbal model into a quantitative one. Quantitative models of habitat construction stretch back to 1996 (Laland et al., 1996), and there are now a variety of other quantitative models (Chisholm et al., 2018; Krakauer et al., 2009; Kylafis & Loreau, 2008; Lehmann, 2008; Scheiner et al., 2021, 2022; Silver & Di Paolo, 2006), along with verbal summaries of the conditions that should favor the evolution of habitat construction (Odling‐Smee et al., 2003, 2013). Thus, the time is ripe to formalize a constitutive theory of the evolution of habitat construction.

We present the domain and propositions for that theory in Table 2. The domain of this theory is environmental conditioning that increases the inclusive fitness of an individual. That conditioning can consist of changes in the state of the environment (e.g., soil processing by earthworms), resource levels, or the creation of artifacts (e.g., nests, sensu Odling‐Smee et al., 2013). That conditioning must be, at least in part, of the abiotic environment. If the effects of the target species are just on other living organisms, that is more properly the domain of theories of co‐evolution. Domains are defined by the nature of the models that they encompass. Models in which the environmental component is strictly abiotic, and therefore cannot also evolve, will be different from those in which other components can evolve. Clearly, however, there is potential overlap in domains if the environment contains both abiotic and biotic components; it is not necessary that theory domains be non‐exclusive and some models can fall into more than one domain.

**TABLE 2:** A constitutive theory of the evolution of habitat construction

Domain: Evolutionary change in the propensity of an individual to directly or indirectly alter its abiotic environment so as to increase its inclusive fitness.
*Propositions:*
1. The state of the environment that would result in the maximal fitness of an individual, group of individuals, or lineage differs from the current state.
2. Individuals are able to alter their environment so as to increase the fitness of themselves or other individuals.
3. The effects of construction on the environment are self‐limiting, either due to feedbacks on the construction process or an upper limit to that construction, and/or are subject to decay.
4. The propensity for construction meets the conditions required for evolution by natural selection.
5. Construction is favored when its benefits are directed at the constructing individual or its’ near kin.
6. Non‐optimal construction may result from costs of construction.
7. Non‐optimal construction may result from trade‐offs between the ability to perform construction and the trait(s) directly affected by the environment.
8. Non‐optimal construction may result from interactions with other processes that alter the fit of an individual to its environment.

All habitat construction models that we are familiar with meet the first four propositions (Table 2). The first two propositions separate instances of adaptive habitat construction from environmental conditioning that is simply a by‐product of other adaptations. The third and fourth propositions are statements about components of the model; the latter suggests that this theory could be considered a subdomain of the constitutive theory of evolution by natural selection (Frank & Fox, 2019). The last four propositions describe conditions that might favor or disfavor the evolution of adaptive construction, and not all may be relevant to all models. The seventh proposition is not relevant to our model. The eighth proposition is not relevant to the version of our model explored here, although it is relevant to other versions (Scheiner et al., 2021, 2022). As with any theory, its components are subject to amendment. We present this constitutive theory in that spirit.

## CONFLICT OF INTEREST

The authors declare no conflicts of interest.

## AUTHOR CONTRIBUTIONS

**Samuel M. Scheiner:** Conceptualization (equal); Formal analysis (lead); Methodology (equal); Software (lead); Writing – original draft (equal); Writing – review & editing (equal). **Michael Barfield:** Conceptualization (supporting); Formal analysis (supporting); Methodology (equal); Software (supporting); Writing – original draft (equal); Writing – review & editing (equal). **Robert D. Holt:** Conceptualization (equal); Formal analysis (supporting); Methodology (equal); Software (supporting); Writing – original draft (equal); Writing – review & editing (equal).

## Data Availability

There are no data to be archived. The program code is available from GitHub: https://github.com/sscheiner1/Plasticity-models/blob/niche20/niche20.txt.
